# Mystery behind the match: an undergraduate medical education–graduate medical education collaborative approach to understanding match goals and outcomes

**DOI:** 10.3402/meo.v21.32235

**Published:** 2016-09-29

**Authors:** Alisa Nagler, Deborah L. Engle, Mariah Rudd, Saumil M. Chudgar, John L. Weinerth, Catherine M. Kuhn, Edward Buckley, Colleen O’Connor Grochowski

**Affiliations:** 1 Division of Education, American College of Surgeons, Chicago, IL, USA; 2 Practice of Medical Education, Duke University School of Medicine, Durham, NC, USA; 3 Office of Curricular Affairs, Duke University School of Medicine, Durham, NC, USA; 4 Office of Continuing Professional Development, Virginia Tech Carilion School of Medicine, Roanoke, VA, USA; 5 Department of Medicine, Duke University School of Medicine, Durham, NC, USA; 6 Department of Surgery, Duke University School of Medicine, Durham, NC, USA; 7 Graduate Medical Education, Duke University School of Medicine, Durham, NC, USA; 8 Department of Anesthesiology, Duke University School of Medicine, Durham, NC, USA; 9 Department of Ophthalmology, Duke University School of Medicine, Durham, NC, USA

**Keywords:** retention rates, home institutions, advanced training, match, graduates, applicants, program quality, medical education

## Abstract

**Background:**

There is a paucity of information regarding institutional targets for the number of undergraduate medical education (UME) graduates being matched to graduate medical education (GME) programs at their home institutions. At our institution, the Duke University, the number of UME graduates matched to GME programs declined dramatically in 2011. To better understand why this decline may have happened, we sought to identify perceived quality metrics for UME and GME learners, evaluate trends in match outcomes and educational program characteristics, and explore whether there is an ideal retention rate for UME graduates in their home institutions’ GME programs.

**Methods:**

We analyzed the number of Duke University UME graduates remaining at Duke for GME training over the past 5 years. We collected data to assess for changing characteristics of UME and GME, and performed descriptive analysis of trends over time to investigate the potential impact on match outcomes.

**Results:**

A one-sample *t*-test analysis showed no statistically significant difference in the number of Duke UME graduates who stayed for GME training. For both UME and GME, no significant changes in the characteristics of either program were found.

**Discussion:**

We created a process for monitoring data related to the characteristics or perceived quality of UME and GME programs and developed a shared understanding of what may impact match lists for both UME graduates and GME programs, leaving the Match somewhat less mysterious. While we understand the trend of graduates remaining at their home institutions for GME training, we are uncertain whether setting a goal for retention is reasonable, and so some mystery remains. We believe there is an invaluable opportunity for collaboration between UME and GME stakeholders to facilitate discussion about setting shared institutional goals.

The National Resident Matching Program^®^ (NRMP^®^ or The Match^®^) provides an impartial venue for coordinating medical students’ preferences for residency positions with program directors’ preferences for applicants (1). Multiple factors influence undergraduate medical education (UME) graduates’ decisions to select a graduate medical education (GME) training program (2–5). Similarly, GME programs are likely to vary greatly across specialties and institutions, but they may also require general criteria and minimal standards that impact how they rank applicants (6–8).

The results of The Match are critical for schools and their graduates, as well as for GME programs and their sponsoring institutions. For medical schools, the success of graduates helps shape the school's reputation and the consequent student caliber that it attracts. For GME programs, The Match is an important variable in assessing program quality, with success typically defined as filling available positions with top-ranked candidates. The Match is used as a recruitment tool, identifying GME programs as able to attract high-caliber applicants who mutually agree on fit. Since many institutions are home to both school of medicine and GME training programs, one could imagine an institution leveraging its resources to train doctors longitudinally – UME to GME – and perhaps even ‘growing’ their own faculty.

In 2011, the number of Duke University UME graduates being matched to Duke GME programs dropped by nearly half; we sought to investigate the reason for this change. We do not have a stated goal for retaining graduates in Duke's GME programs; nevertheless, we asked the following questions: 1) Did the characteristics of our graduates change so that GME programs did not rank them highly?; or (conversely) 2) What happened to our GME programs that would have caused graduates to rank them lower?

Prompted by these questions, a research team comprising members from UME and GME programs set out to identify what might have contributed to this decline. Our objectives were to identify variables that can be used to assess the characteristics or perceived quality of UME graduates and GME training programs and to determine whether these characteristics have an impact on match outcomes.

The purpose of this article is to share the usefulness of the variables and the process we used, so that other institutions may be able to replicate in some form this innovation and learn from their own data. Actual Match outcome data can be quite sensitive and are not often shared throughout an institution, let alone outside of an institution. Both medical schools and GME programs may be concerned that these outcomes will taint their reputations. Without sharing actual Match outcomes, the authors attempt to uncover some of the Match mystery, suggesting opportunities may be lost with such secrecy and no open dialogue. The Match is clearly a high-stakes and high-visibility phenomena – one to which we hope to bring the potential for deliberate planning and transparency.

## Methods

An initial review was conducted of the longitudinal trend in the number of Duke University UME graduates staying for GME training. Specifically, we calculated the mean number of UME graduates staying for GME training across a 12-year time period (2001–2012). We then used a one-sample *t*-test to determine whether this 12-year mean was significantly different from the number who matched in 2011. The level of statistical significance was set at *p*<0.05.

From there, we attempted to determine whether the characteristics of UME students and/or GME programs changed over time. As can be seen from the results section, actual outcomes are not included. Instead the article is intended to share the process used to analyze the Match outcomes in a way that proved very meaningful to one institution and could be replicated by others.

### Identification of variables

We identified variables to assess the UME and GME characteristics at our institution (Table 1), as well as the processes and parties responsible for collecting the relevant data. Data were collected, and descriptive statistics were calculated for each of the variables described below. The Duke University School of Medicine Institutional Review Board approved this project.

**Table 1:** Individual and institutional characteristics

Question/category	Variables that may measure quality and impact match results	Assumption
Characteristics of Duke	MCAT score	Marker of potential success as student
SOM students	% underrepresented minorities in medicine	Increased class diversity contributes to increase in quality of educational program
	Step 1, Step 2 CS, Step 2 CK (% pass)	Used as screening tool by program directors
	Clerkship grades	Used as screening tool by program directors
	Third year – external scholarship	Competitively awarded; infers highly functioning student
	Third year – away research	Accepted at another school; infers student is highly regarded by another school
	Fourth year – away electives	Assume away electives are done for audition purposes
	Match outcomes	Match =intended SOM outcome
Characteristics of Duke	Accreditation cycle length	Full length contributes to reputation as strong program
GME programs	Depth of rank list to fill program	Less depth infers ability to attract top choices more quickly/efficiently/easily
	Competing institutions	If not Duke, SOM graduates would choose/end up at peer institutions
	USMLE Step 3 (% pass)	Score is a measure of resident's knowledge and ability
	Medical schools of residents	Program directors consider ranking of applicant's SOM when making selection – more likely to choose applicant from higher-ranked school
	Diversity in GME programs (% underrepresented minorities in medicine)	SOM graduates may prioritize GME programs according to level of diversity
Specialty choice trends	NRMP data	National trends in specialty choice impact match outcomes
GME to faculty	What % SOM graduates stay for GME; GME graduates stay on for faculty?	When graduates decide to stay at training institution, it speaks to quality and reputation of the institution

GME, graduate medical education; MCAT, Medical College Admission Test; NRMP, National Resident Matching Program; SOM, school of medicine; Step 2 CK, Step 2 Clinical Knowledge; Step 2 CS, Step 2 Clinical Skills; USMLE, United States Medical Licensing Examination.

### Characteristics of UME students

In order to understand whether characteristics of Duke UME graduates changed over time, we identified standardized exams and variables that are often used as indicators of student success and for which there is an existing benchmark data (Table 1).

### Pre-matriculation metrics

Pre-matriculation metrics included Medical College Admission Test (MCAT) scores and demographics of each class. The percentage of underrepresented minority in medicine (URMiM) demographic was chosen because we were curious whether class diversity had been maintained. Duke upholds the value that class diversity contributes positively to the quality of the educational program and the experience of its students.

### Performance metrics

Clerkship grades, United States Medical Licensing Examination^®^ (USMLE^®^) Step 1 scores, Step 2 Clinical Knowledge scores, and Step 2 Clinical Skills pass rates were also included in the analysis because they provide national benchmarks for comparison.

### The Match outcomes

We also included students’ Match outcomes. For each UME graduate, we tracked the residency program and corresponding institution to which he or she was matched. We also tracked the number of students who were unsuccessful in The Match process.

### Characteristics of GME programs

In order to understand whether characteristics of Duke GME programs changed over time, we identified variables of training-program quality that are informally accepted as benchmark standards. Some of these variables include characteristics that existing literature (2–5) suggests UME graduates should consider when determining their rank list (Table 1).

### Accreditation cycle length

When this project was initiated, GME program cycle length was a national accreditation metric for quality and was available for sponsoring institutions through the Accreditation Council for Graduate Medical Education (ACGME). The authors note that with the Next Accreditation System (NAS), accreditation cycle length is no longer calculated or used and, therefore, would be replaced by ‘program status’ going forward (9).

### Rank list

The Match results were collected and analyzed to identify how far down the rank list programs went to fill their class quota. This analysis is controversial given the multitude of ways that programs formulate their match lists. Nonetheless, we thought this information was an important metric to include because it might provide some insight with regard to longitudinal trends within programs.

### Competing institutions

We identified the matched institution for Duke UME students who were ranked above the last spot filled, but who did not end up at Duke. We believed it was important to know which institutions these students ranked more highly than Duke because it would be reassuring to know these students were training at competitive peer institutions, even if ‘competitive’ was subjectively determined.

### USMLE

Duke's GME policy requires residents to pass USMLE Part 3 before entering their third postgraduate year. Any dips or rises in average USMLE scores may indicate issues of resident or program quality. Both average scores and national pass rates were collected.

### UME medical schools of those in Duke GME programs

With the emphasis on undergraduate and medical school rankings, we collected and analyzed the medical schools attended by those in Duke GME programs. While there may be controversy regarding whether graduates from highly ranked schools are better trained, this information may be perceived as an indicator of GME program quality.

### Percentage of underrepresented minorities

We recognize that there may be varied interpretations of the outcome of this variable. We compared it with the national norm and relied on our value that diversity among our learners contributes to the quality of the educational program. Applicants may prioritize GME programs according to the existing level of diversity among the residents and faculty of the program.

### Timeframe and programs

We delimited our analysis to the years 2010–2014 and to three large training programs: internal medicine, general surgery, and diagnostic radiology. With close to 100 GME programs and more than 1,000 residents and fellows, it was important to narrow the analysis so we could examine in-depth details of a number of variables. The analysis could easily be replicated and include any GME programs but for purposes of identifying meaningful variables and collecting and organizing data, we started with just three large GME programs. These three programs were purposely included since the Duke University School of Medicine and GME leadership agreed they provided a good characterization of matching history for the institution and would offer sufficient representative data for a meaningful analysis.

## Results

Though there were fluctuations over time, a one-sample *t*-test revealed no significant differences (*p*>0.05) in match rates, as the average retention rate over 12 years was approximately 21 matches. Yet in 2011, the match rate dropped by almost half, down to 12. In 2012, the match rate rebounded back to 21 and have sustained at that approximate rate.

Data about Duke undergraduate medical school students who applied to Duke GME programs were divided into three categories: those above the fill line who were matched at Duke, those above the fill line who were not matched at Duke, and those below the fill line. Meaning, if a GME program sought to enroll five medical school graduates and ranked 20, those ‘above the fill line who are matched at Duke’ are those who the Duke GME program ranked high and also Duke was high on the graduates’ rank list, and thus they ended up at Duke. Those ‘above the fill line who were not matched at Duke’ are those who Duke ranked high, but the graduate ranked another institution higher than Duke (who also ranked him/her high). Those ‘below the fill line’ are those whom Duke didn't rank high enough to come to Duke. Meaning, the Duke GME program filled its class of five before getting to these other individuals on the rank list. For each category, we identified the rank of each student on the Duke GME program match list and the confirmed training institution if they did not end up at Duke. These data were organized in a table format to best illustrate the categories. Each section had a de-identified list of individuals in any of the three designated programs (internal medicine, diagnostic radiology, or general surgery) for each of the 5 years of data analyzed (Table 2). (As noted above, given the sensitive nature of The Match outcomes, the actual data are not included here but instead the format for organizing the data for easy review and analysis is provided). The data were also displayed in a bar graph format (not shown), with the numerator being the number of spots for programs to fill and the denominator being how far down the rank list programs went to fill those spots. This bar graph provided a clear depiction of these data: the closer to 1, the more ‘successful’ the match.

**Table 2:** Sample table created to evaluate match outcomes

Ranked above fill line: came to Duke		

Program	Rank #	GME training institution
Internal medicine		Duke
Diagnostic radiology		Duke
General surgery		Duke

Ranked above fill line: did not come to Duke		

Program	Rank #	GME training institution

Internal medicine		
Diagnostic radiology		
General surgery		

Ranked below fill line		

Program	Rank #	GME training institution

Internal medicine		
Diagnostic radiology		
General surgery		

GME, graduate medical education.

For those Duke UME graduates who did not end up at Duke for GME training, but had been ranked highly by Duke (above the fill line), data were also collected on where they were matched. While somewhat subjective, we were interested in whether these individuals ended up at peer institutions for their training. Our analysis suggested that was the case as these individuals were matched at GME programs of seemingly like quality and caliber. These are the institutions Duke compares itself with, and academic advisors recommend as similar GME training programs.

Upon reviewing variables identified for both UME and GME, there was no evidence to indicate a major change (for better or worse) over time in any of the areas. Nothing from the analysis suggested a fluctuation in the quality of the UME or GME programs, that could explain the drop in the number of Duke University UME graduates being matched to Duke GME programs. A brief descriptive summary of our findings (by variable) can be found in Table 3.

**Table 3:** Summary of UME–GME match analysis

UME	GME
Matriculant demographic Trends stable over time	Accreditation cycle Above national average across research period
Clerkship grades Trends stable over time	Depth of rank list to fill program Trends stable over time
USMLE scores Above or at national mean; trends show increasing scores over time	Where did applicants go if not Duke? Institutions with similar rigor and reputation
Third year Trends stable over time; dip in number of scholarships awarded in 2011 (fewer scholarships available that year)	USMLE results Above national pass rate of 94% (for 2010)
Fourth year # of away electives slightly trending upward over time	Diversity in GME programs Opportunity to come closer to meeting national average of underrepresented minorities in medicine (stable over time)

GME, graduate medical education; USMLE, united states medical licensing examination; UME, undergraduate medical education.

## Discussion

We set out to determine whether there was something specific that contributed to the fluctuation in Duke UME graduates who remained at Duke for their GME training. We did not find specific factors that explained or predicted the decline in 2011. A thorough analysis of the individual and institutional characteristics included in this study revealed that they have been fairly stable over time. However, we did learn several lessons as a result of this collaboration and identified variables worth considering when analyzing our Match results.

First, we realized that the UME and GME programs were using different criteria for calculating the number of graduates staying at Duke for GME training. For example, what about learners doing their preliminary year elsewhere but then returning to Duke for training, or those only staying for a preliminary year but then leaving for their primary program? We had to reach a consensus to ensure data were consistent between UME and GME programs, as well as for future data collection and analysis.

Second, we learned valuable lessons in terms of identifying which data to collect and how to organize these data for presentation. There were numerous conversations with individuals across UME and GME regarding what information would be useful in trying to understand the characteristics of those graduating from our medical school, and those applying to our residencies. We also found presentation formats that helped increase the understanding and consumption of data regarding match outcomes, such as those in Table 2.

Third, our project brought to light the question of whether there should be an institutional target related to the number of UME graduates who stay for GME training. The leadership at Duke University School of Medicine and Duke University Hospital are considering this question, and their insights will provide additional clarity.

Finally, this study allowed us to identify critical variables for which we can continue to collect and track data. A process is now in place, which will allow for ongoing evaluation over time with the ability to monitor progress toward an ultimate goal, once determined. Individuals in both the UME and GME offices collect and organize data in each of the variables discussed above and present collaboratively to interested parties annually. Fluctuation in any of the variables over the years provides an opportunity to further examine the state of both UME and GME. The findings provoke conversation about the UME and GME goals and have led to discussions about changes in program and institutional level practices. Examples have included processes for creating GME program rank list, discussions around medical student opportunities to study for USMLE, and preparing students for GME program interviews.

Our study did have a couple of limitations. First, the data collected were the objective data available; they did not include graduates’ experiences during the GME interview process, which is known to be an important factor in decision-making (2). Second, we were limited in the longitudinal nature of our investigation because variables that may be important are difficult to measure (e.g., a single encounter with someone, geography, and the subjective opinion of an institution).

## Conclusion

This study proved to be a valuable experience in collaboration between Duke UME and GME, bringing into focus a question that had not previously been explicitly asked: What is the ideal retention of UME graduates at a given institution? We learned the value of defining *a priori* what factors should be and are evaluated, including an awareness that not all relevant data come through the NRMP. We began the process of talking openly about The Match at our institution with the goal of furthering individual and institutional interests of UME and GME. The variables identified to better understand UME graduate and GME applicant characteristics, and the process used as described above should prove meaningful to other institutions interested in opening the dialogue and considering more deliberate planning related to The Match.

## Previous presentations

***Posters:***

Exploring the fluctuation in number of Duke School of Medicine graduates who matched in Duke Graduate Medical Education Programs (Edward Buckley MD, Colleen O'Connor Grochowski PhD, Alisa Nagler JD EdD, Deborah Engle EdD, Mariah Rudd, John Weinerth MD) Research in Medical Education (RIME) at American Association of Medical Colleges Annual Meeting, Philadelphia, PA, November 2013.

Exploring the fluctuation in number of Duke School of Medicine graduates who matched in Duke Graduate Medical Education Programs (Edward Buckley MD, Colleen O'Connor Grochowski PhD, Alisa Nagler JD EdD, Deborah Engle EdD, Mariah Rudd, John Weinerth MD) American Association of Medical Colleges Southern Group on Educational Affairs. Miami FL, March 2014.

***Presentations:***

Exploring the fluctuation in number of Duke School of Medicine graduates who matched in Duke Graduate Medical Education Programs (Edward Buckley MD, Colleen O'Connor Grochowski PhD, Alisa Nagler JD EdD, Deborah Engle EdD, Mariah Rudd, John Weinerth MD) Accepted for oral presentation for Research in Medical Education (RIME) at American Association of Medical Colleges Annual Meeting, Philadelphia, PA, November 2013.

Unveiling the mystery behind the match (Colleen O'Connor Grochowski PhD, Alisa Nagler JD EdD, Deborah Engle EdD, Mariah Rudd, Saumil Chudgar MD) Association of American Medical Colleges – Southern Group on Educational Affairs Meeting. Charlotte NC, April 2015.
